# Association between maternal marginalization and infants born with congenital heart disease in Ontario Canada

**DOI:** 10.1186/s12889-023-15660-5

**Published:** 2023-04-28

**Authors:** Qun Miao, Sandra Dunn, Shi Wu Wen, Jane Lougheed, Phoebe Yang, Michael Davies, Carolina Lavin Venegas, Mark Walker

**Affiliations:** 1 Better Outcomes Registry & Network (BORN) Ontario, Children’s Hospital of Eastern Ontario, Ottawa, ON Canada; 2 Children’s Hospital of Eastern Ontario Research Institute, Ottawa, ON Canada; 3 School of Epidemiology and Public Health, Faculty of Medicine, University of Ottawa, Ottawa, ON Canada; 4 Department of Public Health Sciences, Queen’s University, Kingston, ON Canada; 5 Clinical Epidemiology Program, Ottawa Hospital Research Institute, Ottawa, ON Canada; 6 School of Nursing, University of Ottawa, Ottawa, ON Canada; 7 Department of Obstetrics & Gynecology, University of Ottawa, Ottawa, ON Canada; 8 Department of Pediatrics, Children’s Hospital of Eastern Ontario, Ottawa, ON Canada; 9 Department of Pediatrics, University of Ottawa, Ottawa, ON Canada; 10 Adelaide Medical School, Faculty of Health and Medical Sciences, the Robinson Research Institute at the University of Adelaide, Adelaide, South Australia 5005 Australia; 11 International and Global Health Office, University of Ottawa, Ottawa, ON Canada; 12 Department of Obstetrics, The Ottawa Hospital, Gynecology & Newborn Care, Ottawa, ON Canada

**Keywords:** Retrospective cohort study, Mother, Pregnancy, Infant, Congenital heart disease, Socioeconomic status, Ontario Marginalization Index

## Abstract

**Background:**

This study aims to evaluate the impact of socioeconomic status (SES) on the risk of congenital heart disease (CHD) since previous studies have yielded inconsistent results.

**Methods:**

We conducted a population-based retrospective cohort study, including all singleton live and still births in Ontario hospitals from April 1, 2012, to March 31, 2018. We used linked records from the Better Outcomes Registry & Network Information System, the Canadian Institute for Health Information databases, and the Ontario Marginalization Index (ON_Marg). ON_Marg was estimated at a dissemination area level using Canadian Census 2016 data and categorized into quintiles. Multivariable logistic regression models were performed to examine the relationships between four ON_Marg indices (material deprivation, dependency, ethnic concentration, residential instability), as proxies for maternal SES and the risk of infant CHD. We adjusted for maternal age at birth, assisted reproductive technology, obesity, pre-existing health conditions, substance use during pregnancy, mental health conditions before and during pregnancy, rural residence, and infant’s sex in the analysis.

**Results:**

Among the cohort of 776,799 singletons, 9,359 infants had a diagnosis of CHD. Of those, 3,069 were severe CHD and 493 cases were single ventricle CHD. The prevalence of all infant CHD types was higher for males relative to females. Compared to mothers living in neighbourhoods with the lowest material deprivation, mothers with highest material deprivation had a 27% (adjusted OR = 1.27; 95% CI: 1.18–1.37) higher odds of having an infant diagnosed with CHD. Mothers living in neighbourhoods with the highest minority ethnic and immigrant concentration tend to have infants with 11% lower odds of CHD (adjusted OR = 0.89; 95% CI: 0.82–0.97) as compared to those living in the least ethnically diverse communities. Maternal dependency and residential stability quintiles were not significantly associated with the risk of CHD.

**Conclusion:**

Higher maternal material deprivation was associated with increasing odds of infant CHD, whereas neighbourhood minority ethnic concentration was inversely associated with the odds of infant CHD. Our study further confirms that poverty is associated with CHD development. Future investigations might focus on the causal pathways between social deprivation, immigrant status, ethnicity, and the risk of infant CHD.

**Supplementary Information:**

The online version contains supplementary material available at 10.1186/s12889-023-15660-5.

## Introduction

Congenital heart disease (CHD) is a common type of birth defect that affects the heart’s structure [1] causing more than 180,000 deaths globally per year in infants younger than one year old [2] and is a major cause of life-long disability. With a global prevalence of 15.9 to 19.9 cases per 1,000 births [2], CHD accounts for 30 to 45% of all congenital anomalies [3, 4]. The global prevalence of CHD has steadily increased by 4.2% since 1990 [2] which can be partially attributed to improved prenatal screening and diagnosis of fetal CHD [5, 6]. In Canada, the CHD prevalence is 12.3 per 1,000 total births [7]; with an annual birth rate of around 358,000 births [8], this corresponds to 4,400 infants born in Canada with CHD every year. Together with rising costs related to care of infants with CHD including hospitalization, the burden of CHD represents a major public health issue in Canada [9, 10].

Although various epidemiological studies have been conducted, the majority of CHDs are of unknown etiology [1, 11–19]. Certain genetic, environmental and other factors contribute to the development of CHD [1, 11]. Known genetic factors include chromosomal aneuploidies and single gene defects [11], whereas known non-genetic factors include advanced maternal age [12, 15], rubella virus infection [12] and exposure to environmental hazards during pregnancy [3, 13, 15], pre-pregnancy maternal obesity [13], the use of assisted reproductive technology (ART) [13], maternal exposures to social drugs [13], cigarette smoking [13], alcohol [12, 13], and certain prescribed medications [12], and maternal pre-existing and gestational diabetes [11–15]. In addition, studies have suggested that there may be sex variation in the risk of CHD, although the findings are inconsistent [13–16].

Furthermore, recent studies have identified disparities in the prevalence of CHD in infants by maternal race and socioeconomic status (SES) [17–20]. Specifically, studies have shown that lower household income, lower maternal education, unemployment status, social isolation, and certain racial groups were associated with an increased risk of having an infant born with CHD [19–22]. Similarly, in a retrospective cohort study conducted in the United Kingdom, it was observed that the CHD incidence rate ratio was significantly higher in infants of Asian (IRR = 1.5; 95% CI: 1.4–1.7) and Black (IRR = 1.4; 95% CI: 1.3–1.6) ethnicities as compared to White infants; moreover, children of non-White race groups were more likely to live in deprived postal code regions compared to White children [23].

Researchers have also found other social factors to be associated with adverse maternal and birth outcomes [17]. For example, one prospective cohort study conducted in the United States with 3,428 mother–infant pairs found that there were statistically significant associations between severe housing insecurity during pregnancy and low birth weight and preterm birth (RR = 1.75 95% CI: 1.28–2.32) [24].

Despite multiple studies examining the association between maternal SES and CHD, results were inconsistent across the different geographical areas and various study populations. For example, one meta-analysis of two ecological, seven case–control, and two cohort studies did not find any significant associations between neighborhood SES variables and the risk of CHD [25]. Reasons for these observed inconsistencies could be linked to the use of different SES indicators to measure maternal SES in different populations and geographical areas [26, 27]. However, this may be expected as SES is a multi-dimensional construct, and no single SES indicator can capture all aspects of one individual’s socioeconomic position [26]. In addition, SES factors may intersect with each other to depict a person’s social position at a specific point in the lifespan [26], therefore, composite SES indicators might be a better proxy of SES measurement [28, 29]. When individual-level data is unknown, area-based deprivation indices can be proxies of individual SES indicators [27–30].

Ontario researchers have developed the Ontario Marginalization Index (ON_Marg), which includes four composite SES dimensions: material deprivation, dependency, residential instability, and ethnic concentration [31]. ON_Marg can be used for surveillance and research surrounding health inequities [32]. To date, over 70 studies have used ON_Marg to evaluate social determinants of health by geographical area and the relationship with a wide range of health outcomes and health system inefficiencies in Ontario [33, 34]. While the ON_Marg has been used to assess inequities in maternal and childcare research [35, 36], no studies have used the index to explore its association with congenital birth defects, in particular the association between maternal SES and infant CHD. In this study, we aim to use multiple area level SES factors to further examine the relationships between the various dimensions of maternal SES and the risk of CHD and to explore the sex variation in these associations.

## Methods

### Study aim

This study aims to examine the association between maternal SES and the risk of CHD among infants.

### Study design

This was a population based retrospective cohort study analyzing population-level data from Ontario between April 1^st^, 2012, to March 31^st^, 2018.

### Study population

This study cohort consisted of all singleton live births, stillbirths, and late-stage pregnancy terminations with birthweight ≥ 500 g or a gestational age ≥ 20 weeks that occurred in Ontario hospitals. We excluded multiple gestational births and births where the maternal residence was outside of Ontario, Canada.

### Data sources and data linkage

BORN databases: Better Outcomes Registry & Network (BORN) is a registry that collects data on every pregnancy and birth in Ontario through the BORN Information System (BIS) [37–39]. The BORN prenatal databases capture maternal demographic characteristics and health behaviors; pre-existing maternal health problems; prenatal screening; obstetric complications; intra-partum interventions; fetal anomalies and birth outcomes in pregnancy; labour and birth, and postpartum stages [37, 39]. The data is collected by individual encounters but also aggregated into maternal pregnancy and infant ‘course of care’ datasets for individuals [37, 40]. Datasets in the BIS were used to perform the analysis including aggregate pregnancy, aggregate infant, antenatal specialty (AS) for high-risk pregnant women clinics, prenatal screen, and prenatal screening follow-up (PSFU) data [22]. BORN has strived to ensure high data quality in the BIS through an ongoing data validation process [39, 41], quality checks, and formal training sessions for individuals entering data [40]. A number of papers and reports have been published using these data [37–41].

Canadian Institute for Health Information (CIHI): The Discharge Abstract Database (DAD) and the National Ambulatory Care Reporting System (NACRS) are run and maintained by CIHI [42, 43]. Each year, BORN receives CIHI-DAD and CIHI-NACRS maternal, newborn, and child (up to one year of age) records from acute care and emergency facilities in Ontario [39]. By using these data sources in conjunction with the BIS, we are able to identify infants who had a diagnosis of CHD in hospital up to one year of age [22].

The ON_Marg was developed by Public Health Ontario in collaboration with MAP Centre for Urban Health Solutions and St. Michael’s Hospital [31]. Following a literature review of the 2001 Canadian Census of Population, 42 preliminary variables related to marginalization and health inequities were selected [31]. Subsequently, a principal component factor analysis was conducted (Eigenvalues > 1) to reduce it to 18 indicators spread across four dimensions of marginalization: material deprivation, dependency, residential instability, and ethnic concentration [31]. The 2016 Index was created from 2016 Census data on 20,640 dissemination areas (DAs) [31]. Within each dimension, factor loading was used to create an asymmetrically standardized index and each dimension of marginalization was sorted and broken down into five equal sized quintiles (Q1 = least marginalized, Q5 = most marginalized) [31].

The Postal Code Conversion File Plus (PCCF +) version 7B was developed by Statistics Canada, and it contains the most updated postal codes and their corresponding geographic DAs, the smallest standard geographical area in Canada from Canada Census 2016 [44, 45]. By linking the study cohort and PCCF + using maternal residential postal codes, and then linking the cohort with ON_MARG data using unique DA IDs, we can obtain maternal neighborhood level ON_Marg at a DA level.

**Fig. 1:**
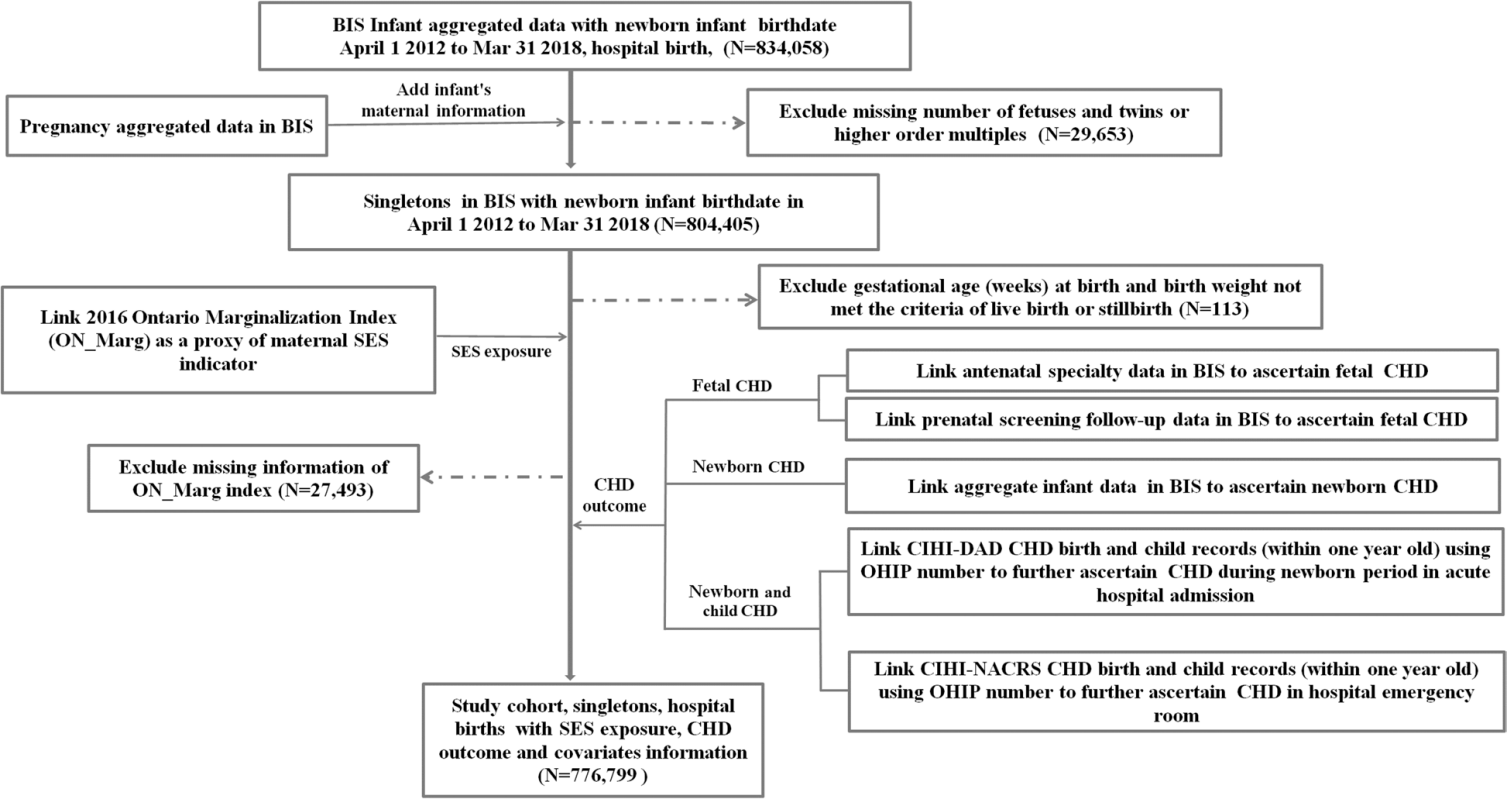
Flowchart of data sources and data linkage for study cohort (April 1 2012—March 31 2018)

We started to perform the linkage process within the BIS system. The study cohort was obtained from the aggregate infant data of birthdates within the inclusion timeframe. This dataset was linked to the aggregate maternal pregnancy data to obtain maternal information. The cohort was further linked to the AS and PSOF encounter data in the BIS, CIHI-DAD and CIHI-NACRS databases to define the outcome, CHD. The outcome of CHD, ON_MARG (composite SES indicators exposures) and covariates were obtained from multiple data sources. Please see Fig. 1 for the data linkage flow chart.

### SES measurement using Ontario Marginalization Index

The ON_Marg, derived from 2016 Canada Census, includes four dimensions, which are material deprivation, dependency, residential instability and ethnic concentration [31]. Each index was categorized into quintiles on a DA level in Ontario [31]. DA is a small geographical area, which has been considered to be relatively stable [46]. The average population of a DA ranges from 400 to 700 persons [46]. Material deprivation represents poverty and the (in)ability to purchase basic material needs [31]. This index was developed from five Census variables: percentage of single parent families, percentage of individuals below the low-income cut-off and who receive income from government transfer payments, percentage of housing that needs major repair, percentage of unemployment of those aged 15 + , and percentage of individuals aged 25 + who do not have a secondary or postsecondary degree [31]. Dependency calculates the proportion of individuals who do not receive an income from employment and who rely on the income of others [31]. This index was calculated using three indicators: percentage of seniors aged 65 or older, dependency ratio, and labour force participation from those aged 15 + . Residential instability is closely tied to housing and family instability [31]. This index was developed based on seven Census variables: percentage of individuals living alone, average number of persons per dwelling, percentage of single/divorced/widowed, percentage of dwelling not owned, percentage of multi-unit housing, percentage of non-youth populations, and percentage of residential mobility [31, 46]. Ethnic concentration measures populations who may experience marginalization [31] and includes two indicators: recent immigrants and percentage of visible minorities living within an area [31]. Refer to Appendix A to see the detailed table describing the indicators for each index.

### Outcomes

All CHD cases captured in the prenatal stage were identified from the AS and PSFU datasets. Newborn diagnoses for CHD are collected in the birth child, postpartum child, and neonatal intensive care encounters of the BIS, and are aggregated into one infant dataset, which is the one we used to capture newborn CHD. To capture additional newborn CHD and CHD diagnosed during the first year of infancy we used the CIHI-DAD and CIHI-NACRS databases. In the BIS, CHD was coded in an anomaly picklist based on clinical diagnosis. In CIHI datasets, CHD was coded using the International Statistical Classification of Diseases and Related Health Problems, 10th Revision, Canadian adaptation (ICD-10-CA). CHD was divided into three types of outcomes: overall CHD (yes vs. no), severe CHD (yes vs. no) and single ventricle CHD (yes vs. no). Single ventricle CHD is a type of CHD but is more severe. Please see details on CHD classifications and grouping of CHD types in Appendix B.

### Covariates

The covariates included maternal age at delivery, ART conception, obesity, pre-existing maternal health condition during pregnancy, mental health status illness in pre-pregnancy and during pregnancy, social drug intake, alcohol consumption and smoking status during pregnancy, maternal residence in urban or rural area and infant sex [19, 22].

### Statistical analysis

We conducted a descriptive analysis showing the distributions of maternal and infant characteristics by overall CHD, severe CHD, single ventricle CHD and all study population. Multivariable logistic regression models were performed to examine the relationships between the maternal ON_Marg and the risk of overall CHD while considering covariates including maternal age at birth, ART, obesity, pre-existing health conditions, substance use during pregnancy, mental health status illness in pre-pregnancy and during pregnancy, rural or urban maternal residence, and infant’s sex. An interaction test was performed to evaluate the effects of infant sex on the relationships between four ON_Marg indices and the risk of overall CHD infants. The crude and adjusted odds ratios (OR) and 95% confidence intervals (CI) were calculated. Since CHD is a rare disease (less than 10% of prevalence), ORs were used to estimate RRs. Further analysis was conducted including types of CHD prevalence and the associations of ON_Marg index with the risk of infant CHD. We used SAS 9.4 (SAS Institute Inc., Cary NC) to perform all data linkages, data management, and data analysis.

## Results

A total of 776,799 singleton births born in Ontario hospitals from April 1^st^, 2012, to March 31^st^, 2018, were identified and included in the final analysis. Among them, 9,359 (12.1 per 1,000 births) infants were diagnosed as CHD, 3,069 (4 per 1,000 births) as severe CHD and 493 cases (0.6 per 1,000 births) as single ventricle CHD.

Table 1 displays maternal and infant characteristics of this study cohort of the entire population and by CHD status (overall CHD, severe CHD, and single ventricle CHD). There were higher percentages of obesity, maternal age ≥ 35, ART derived pregnancy, smoking, alcohol consumption and recreational drug use during pregnancy, pre-pregnancy maternal health conditions, and mental health illness in pre-pregnancy and during pregnancy in mothers of infants with CHD compared to those in the entire study population. The percentages of pregnant people with pre-existing maternal health conditions and mental health illnesses in pre-pregnancy or during pregnancy were higher in mothers of infants born with severe CHD and single ventricle CHD as compared to overall CHD status.

**Table 1:** Distributions of maternal characteristics among different types of CHD and overall populations

	**Overall CHD**		**Severe CHD**		**Single ventricle CHD**		**All population**	
	*N*	Col %^a^	*N*	Col %^a^	*N*	Col %^a^	*N*	Col %^a^
**Maternal pre-pregnancy body mass (mean ± SD) index (BMI) in kg/m**^**2**^**, mean ± SD**	25.05 ± 6.16		25.98 ± 6.71		26.10 ± 6.29		26.08 ± 6.87	
**Maternal age at birth in years, mean ± SD**	30.94 ± 5.73		31.16 ± 5.77		30.76 ± 5.87		30.62 ± 5.29	
< 30	3663	39.18	1160	37.82	204	41.46	312,222	40.22
30–34	3101	33.17	1015	33.90	159	32.32	283,473	36.52
35 +	2584	27.64	892	29.08	129	26.22	180,591	23.26
**Obesity, BMI ≥ 30 kg/m**^**2**^								
No	6310	67.42	614	68.72	99	70.59	565,731	72.83
Yes	1857	19.84	2109	20.01	348	20.88	118,179	15.21
Missing	1192	12.74	346	11.27	46	9.33	92,889	11.96
**ART derived pregnancy**								
No	8983	95.98	125	95.93	20	95.94	752,106	96.82
Yes	376	4.02	2944	4.07	473	4.06	24,693	3.18
**Maternal smoking or social drug use or alcohol consumption during pregnancy**^b^								
Yes	1444	16.06	454	15.48	69	14.84	89,496	11.81
No	7545	83.94	2479	84.52	396	85.16	668,438	88.19
Missing	370		136		28		18,865	
**All types of mental health illness in pre-pregnancy or during pregnancy**								
Yes	1831	19.56	595	19.39	102	20.69	114,373	14.72
No	7528	80.44	2474	80.61	391	79.31	662,426	85.28
**Pre-pregnancy health conditions**								
Yes	2772	29.62	1059	34.51	184	37.32	143,977	18.53
No	6587	70.38	2010	65.49	309	62.68	632,822	81.47
**Pregnancy outcome**								
Stillbirth^c^	353	3.77	242	7.89	100	20.28	4710	0.61
Livebirth	9006	96.23	2827	92.11	393	79.72	772,089	99.39
**Termination**								
Yes	289	3.09	210	6.84	95	19.27	1537	0.20
No	9070	96.91	2859	93.16	398	80.73	775,262	99.80
**Rural residence**								
Yes	1098	11.73	331	10.79	50	10.14	84,346	10.86
No	8261	88.27	2738	89.21	443	89.86	692,453	89.14

^a^ % calculation represents the column percentage

^b^ Missing values were excluded for % calculation

^c^ Include fetal deaths due to spontaneous loss or termination at ≥20 gestational weeks or fetus birth weight ≥500 grams

Table 2 shows distributions of the ON_MARG’s four-dimension quintiles among types of CHD and overall infant populations. For the material deprivation, a higher percentage of infants with CHD had mothers living in the most materially deprived neighbourhoods (Q5) as compared to other quintiles. Similarly, there was a higher percentage of infants with CHD whose mother lived in the most ethnically diverse neighbourhoods (Q5) as compared to other quintiles. Lastly, for the dependency indicator, an opposite trend was observed. There was a lower percentage of infants with CHD born to mothers who were in the most dependent neighbourhoods (Q5) compared to other quintiles.

**Table 2:** Distributions of Ontario Marginalization Index among different types of CHD and all infant populations

	**Overall CHD**		**Severe CHD**		**Single ventricle CHD**		**All population**	
	*N*	Col %^a^	*N*	Col %^a^	*N*	Col %^a^	*N*	Col %^a^
**Ontario Marginalization Index**								
**Material Deprivation**								
1 (least)	1729	18.47	575	18.74	74	15.01	162,581	20.93
2	1708	18.25	553	18.02	96	19.47	150,880	19.42
3	1797	19.2	592	19.29	97	19.68	146,331	18.84
4	1788	19.1	592	19.29	92	18.66	144,239	18.57
5 (Most)	2337	24.97	757	24.67	134	27.18	172,768	22.24
**Dependency**								
1 (least)	3053	33	1004	32.71	150	30.43	261,226	33.63
2	2006	21.43	659	21.47	121	24.54	168,196	21.65
3	1552	16.58	538	17.53	69	14.00	131,924	17.00
4	1390	14.85	451	14.70	69	14.00	114,533	14.74
5 (Most)	1358	14.51	417	13.59	84	17.04	100,920	12.99
**Ethnicity Concentration**								
1 (least)	1306	13.95	376	12.25	65	13.18	99,570	12.82
2	1558	16.65	514	16.75	93	18.86	111,106	14.30
3	1581	16.89	531	17.30	70	14.20	129,421	16.66
4	1869	19.97	626	20.40	89	18.05	165,936	21.36
5 (Most)	3045	32.54	1022	33.30	176	35.70	270,766	34.86
**Residential Instability**								
1 (least)	1922	20.54	614	20.01	93	18.86	170,440	21.94
2	1601	17.11	512	16.68	80	16.23	137,597	17.71
3	1606	17.16	541	17.63	93	18.86	135,740	17.47
4	1832	19.57	595	19.39	95	19.27	142,329	18.32
5 (Most)	2398	25.62	807	26.30	132	26.77	190,693	24.55

^a^ Col % represents the column percentage

To determine the association between each dimension of the ON_Marg index and risk of CHD among infants, the crude and adjusted ORs were calculated and presented in Table 3 with quintile 1 (Q1) being the reference group. Compared to the least materially deprived area, mothers with the highest material deprivation had 27% higher odds of having infants born with CHD. On the contrary, mothers living in neighbourhoods with the highest ethnic concentration had 11% lower odds of having a baby with CHD as compared to the least ethnic concentrated areas. As for the other two indices, dependency and residential instability, there was a slightly higher crude odds of having an infant with CHD in mothers living in the most dependent and residentially unstable neighbourhoods (Q5) as compared to the lowest quintile (Q1); however, after adjusting for other maternal covariates, there were no statistically significant differences.

**Table 3:** Associations between maternal Ontario Marginalization Index and the risk of congenital heart diseases among infants

Variable	Crude OR (CI)	Adjust OR (CI)
**Ontario Marginalization Index**		
**Material Deprivation**		
1 (least)	Reference	Reference
2	1.06 (0.99–1.13)	1.06 (0.99–1.13)
3	1.14 (1.07–1.22)	1.15 (1.07–1.23)
4	1.16 (1.09–1.24)	1.17 (1.09–1.26)
5 (Most)	1.27 (1.19–1.36)	1.27 (1.18–1.37)
**Dependency**		
1 (least)	Reference	Reference
2	1.02 (0.97–1.09)	0.95 (0.89–1.01)
3	1.02 (0.96–1.09)	0.91 (0.85–0.97)
4	1.05 (0.99–1.12)	0.93 (0.86–1)
5 (Most)	1.17 (1.09–1.24)	0.99 (0.92–1.07)
**Ethnicity Concentration**		
1 (least)	Reference	Reference
2	1.07 (0.99–1.15)	1.1 (1.02–1.19)
3	0.94 (0.87–1.01)	0.98 (0.9–1.07)
4	0.85 (0.79–0.92)	0.9 (0.82–0.98)
5 (Most)	0.84 (0.79–0.9)	0.89 (0.82–0.97)
**Residential Instability**		
1 (least)	Reference	Reference
2	1.03 (0.96–1.1)	0.97 (0.9–1.04)
3	1.05 (0.98–1.12)	0.95 (0.88–1.02)
4	1.15 (1.08–1.23)	0.98 (0.91–1.06)
5 (Most)	1.13 (1.06–1.2)	0.99 (0.93–1.07)
**Maternal age at birth**		
< 30 years	Reference	Reference
30–34 years	0.94 (0.89–0.98)	0.99 (0.95–1.05)
> 35 years	1.22 (1.16–1.29)	1.28 (1.21–1.35)
**Obesity (BMI ≥ 30 kg/m2)**		
Yes	1.4 (1.33–1.48)	1.26 (1.19–1.33)
No	Reference	Reference
Missing	1.09 (1.02–1.17)	1.13 (1.06–1.21)
**ART derived pregnancy**		
Yes	1.27 (1.14–1.41)	1.15 (1.03–1.28)
No	Reference	Reference
**Maternal smoking or social drug use or alcohol consumption during pregnancy**		
Yes	1.44 (1.36–1.52)	1.28 (1.2–1.36)
No	Reference	Reference
**All types of mental health illness in pre-pregnancy or during pregnancy**		
Yes	1.44 (1.37–1.52)	1.21 (1.15–1.28)
No	Reference	Reference
**Pre-pregnancy health conditions**		
Yes	1.85 (1.77–1.94)	1.72 (1.65–1.81)
No	Reference	Reference
**Baby sex**		
Male	1.1 (1.06–1.15)	1.1 (1.06–1.15)
Female	Reference	Reference
**Residence in rural area**		
Yes	1.1 (1.03–1.17)	1.04 (0.96–1.12)
No	Reference	Reference

A multivariable logistic regression model was performed to calculate adjusted ORs. All variables are in one model

Adding interaction terms in the multivariable regression model showed that the associations between ON_Marg indices and overall CHD were not significantly different by infant sex (interaction test: *p* = 0.79 for material deprivation, *p* = 0.06 for dependency, *p* = 0.80 for ethnic concentration, and *p* = 0.55 for residential instability). Table 4 displays CHD prevalence by type of CHD and infant sex. For all CHD types, the prevalence of CHD among male infants was significantly higher than for female infants.

**Table 4:** Types of CHD prevalence by infant sex

	**Female** (*n* = 377,395)		**Male** (*n* = 398,612)		
Type of CHD	***N***	**Prevalence**^**a**^	***N***	**Prevalence**^**a**^	***P* value**
Overall CHD	4300	11.4	4996	12.5	< 0.0001
Severe CHD	1388	3.7	1664	4.2	0.005
Single ventricle	212	0.6	275	0.7	0.0243

Of the total population (*n* = 776,799), 792 records had an indeterminate infant sex reported or no infant sex reported, *P*-value from Chi-square test

^a^Prevalence refers to the number of cases per 1,000 births

The analysis results of the associations between ON_Marg indices and risk of CHD stratified by infant sex are shown in Table 5. Mothers living in the most materially deprived neighbourhoods (Q5) was associated with 30% and 25% higher odds of having an infant with CHD (among the male and female infant populations respectively) as compared to mothers living in the least deprived neighbourhoods (Q1). On the other hand, with regard to ethnic concentration, only the female infant specific adjusted odds of CHD remained statistically significant. Male and female infants with mothers living in the most ethnically concentrated neighbourhoods (Q5) had 8% and 14% lower odds of having CHD respectively as compared to infants with mothers living in the least ethnically concentrated neighbourhoods (Q1); the stratum-specific adjusted odds were similar. Sex-stratified adjusted odds of CHD for maternal dependency and residential instability remained statistically insignificant.

**Table 5:** Associations between maternal Ontario Marginalization Index and the risk of congenital heart diseases by infant sex

Female					Male			
**Ontario Marginalization Index**	**Overall CHD**		**Crude OR (95% CI)**	**Adjust OR (95% CI)**	**Overall CHD**		**Crude OR (95% CI)**	**Adjust OR (95% CI)**
	***N***	**Prevalence**^**a**^			***N***	**Prevalence**^**a**^		
**Material Deprivation**								
1 (least)	810	10.2	Reference	Reference	905	10.9	Reference	Reference
2	774	10.6	1.01 (0.92–1.12)	1.02 (0.92–1.13)	923	11.9	1.09 (1–1.2)	1.09 (0.99–1.2)
3	816	11.5	1.1 (1–1.22)	1.11 (1–1.23)	964	12.8	1.18 (1.08–1.3)	1.18 (1.07–1.29)
4	823	11.8	1.13 (1.02–1.24)	1.13 (1.02–1.26)	957	12.9	1.19 (1.09–1.31)	1.2 (1.09–1.33)
5 (Most)	1077	12.8	1.25 (1.13–1.37)	1.25 (1.12–1.39)	1247	14.1	1.3 (1.19–1.42)	1.3 (1.17–1.44)
**Dependency**								
1 (least)	1408	11.1	Reference	Reference	1618	12.1	Reference	Reference
2	926	11.3	1.03 (0.94–1.12)	0.96 (0.88–1.05)	1062	12.3	1.02 (0.94–1.11)	0.94 (0.87–1.03)
3	727	11.3	1.03 (0.94–1.13)	0.92 (0.83–1.02)	818	12.1	1.01 (0.93–1.1)	0.9 (0.82–0.99)
4	590	10.6	0.97 (0.88–1.07)	0.85 (0.77–0.95)	793	13.4	1.12 (1.03–1.22)	0.99 (0.9–1.09)
5 (Most)	649	13.3	1.22 (1.11–1.34)	1.03 (0.93–1.16)	705	13.6	1.12 (1.02–1.22)	0.96 (0.86–1.06)
**Ethnic Concentration**								
1 (least)	593	12.3	Reference	Reference	705	13.7	Reference	Reference
2	722	13.4	1.08 (0.97–1.21)	1.1 (0.98–1.24)	827	14.5	1.06 (0.95–1.17)	1.11 (1–1.23)
3	743	11.8	0.97 (0.87–1.08)	0.97 (0.86–1.1)	836	12.6	0.92 (0.83–1.02)	0.99 (0.88–1.11)
4	843	10.4	0.83 (0.75–0.93)	0.85 (0.74–0.96)	1017	12.0	0.87 (0.79–0.96)	0.94 (0.84–1.06)
5 (Most)	1399	10.6	0.85 (0.77–0.93)	0.86 (0.76–0.98)	1611	11.6	0.83 (0.76–0.91)	0.92 (0.82–1.04)
**Residential Instability**								
1 (least)	866	10.5	Reference	Reference	1039	11.9	Reference	Reference
2	727	10.9	1.04 (0.94–1.15)	0.99 (0.89–1.1)	864	12.2	1.01 (0.93–1.11)	0.95 (0.87–1.05)
3	722	10.9	1.04 (0.94–1.15)	0.94 (0.85–1.05)	872	12.6	1.06 (0.96–1.16)	0.95 (0.86–1.05)
4	857	124	1.19 (1.08–1.31)	1.02 (0.91–1.13)	967	13.2	1.12 (1.02–1.22)	0.95 (0.86–1.05)
5 (Most)	1128	12.2	1.18 (1.07–1.29)	1.03 (0.93–1.14)	1254	12.8	1.09 (1–1.18)	0.96 (0.88–1.06)

^a^Prevalence refers to the number of cases per 1,000 births

## Discussion

In this study, we found the prevalence of CHD in infants as 1.2%. Of the 9,359 infants born with CHD, 32.8% were categorized as severe CHD and 5.27% were diagnosed with single ventricle CHD. The ranges of prevalence on overall CHD, severe CHD and single ventricle CHD were consistent with published reports [4, 47–50].

The material deprivation, and minority ethnic and immigrant concentration indices of the ON_Marg were associated with CHD after adjusting for covariates. Infants with mothers living in the most materially deprived communities (Q5) were associated with a 27% higher risk of CHD as compared to infants with mothers living in the least materially deprived quintile (Q1). On the other hand, infants with mothers living in the most ethnically diverse neighbourhoods (Q5) were associated with a 11% reduce risk of CHD as compared to infants with mothers living in the least ethnically concentrated quintiles (Q1). As for the other two ON_Marg dimensions, dependency and residential instability, no statistically significant differences were observed between the highest and lowest quintiles and the risk of infant CHD after adjusting for other maternal covariates. However, the crude OR for both dependency and residential instability were significant and both dimensions indicated that infants with mothers residing in the highest quintile (Q5) had a slightly higher odds of CHD than those in the lowest quintile (Q1). Furthermore, a higher prevalence of CHD among infants was observed amongst mothers in the highest residential instability quintile (Q5) and the lowest dependency quintile (Q1). After stratifying the association between each dimension of maternal SES and CHD by infant sex, the results resembled the unstratified adjusted odds.

Material deprivation, an indicator of area-based poverty, refers to an individual’s (in)ability to access and obtain basic material needs [31]. It was derived from multiple factors including income, educational attainment, and family structure [31]. The observed link between low maternal material deprivation and higher risk of infant CHD identified in this study is congruent with previous studies conducted in Canada and United States that used other area-based deprivation indicators to examine the relationship between maternal poverty and infant CHD [17, 22, 51, 52]. In our recently published studies, which used birth registry data from BORN Ontario, Canada, lower maternal SES, as indicated by lower educational attainment level, higher unemployment status, and lower household income were positively associated with a 34% (aOR: 1.34; 95% CI [1.24–1.44]), 18% (aOR: 1.18; 95% CI [1.10–1.26]), and 29% (aOR = 1.29; 95% CI [1.20–1.38]) increased risk of having an infant with CHD, respectively [22]. These maternal SES inequities on the impact of risk of CHD were observed in international studies as well [23, 53]. In a population-based cohort study in California, United States, it was found that the incidence of CHD was significantly higher among infants born to mothers who had the lowest neighbourhood SES (OR = 1.31; 95% CI [1.21–1.41]) as compared to those with the highest SES [53]. Similarly, in a population-based retrospective cohort study conducted in Sweden, it was observed that children living in the most deprived neighbourhoods had 20% increase in odds of CHD (adjusted OR = 1.20, 95% CI = 0.99–1.45, *p* = 0.057), compared to those living in the least deprived areas [52].

The finding reinforces the association between families living in poverty and adverse birth outcomes, even in a province with universal healthcare access. Universal access to healthcare does not mean universal use of services in a timely and appropriate manner [54, 55]. Individual and contextual factors influence use and certain families who are deprived, living in poverty, have lower education levels, lack resources, and are under stress may not seek health care for prevention, assessment, and treatment of issues [55]. Poverty reduces the purchasing power of families, which can result in purchasing unhealthy foods, living in poor quality environments, facilitating unfavourable health-related behaviours such as physical inactivity, and underutilizing preventative healthcare services, thereby increasing the risk of CHD [56, 57]. Furthermore, poverty can increase the mother’s stress level and precariousness which might also mediate the risk of CHD through a variety of pathways [57].

The dependency index represents population workforce eligibility (proportion of unemployment) [31]. There is a limited number of studies that have used the ON_Marg dependency index to investigate its link with adverse health outcomes; however, maternal unemployment has been shown to increase the risk of infants born with CHD due to elevated psychological stress and reduced financial capabilities [22, 58, 59]. In contrast to previous findings, no statistically significant relationship between maternal dependency and risk of infant CHD was found in this study after adjusting for other maternal covariates [22]. This could be explained as the dependency index includes the proportion of the population that is 65 years and older; however, mothers are typically between the age of 20 to 40 years old [60]. Therefore, this dimension of the ON_Marg may not reflect maternal SES accurately.

Residential instability quantifies family and housing (in)stability (spatial mobility) and is important as it relates to neighbourhood quality, cohesiveness, and support [31]. As explained by the concept of spatial behavior, individual actions are shaped by where they live [61], so, neighborhood quality is closely tied to employment opportunities and prospects of social mobility and perceived social support [62]. The physical environment is an important physical determinant of health, and poor housing conditions have been shown to negatively impact birth outcomes due to greater exposure to environmental toxins [53, 63–65]. Furthermore, a longitudinal study in the United States found that mothers living in or moving to a disadvantaged neighbourhood had lower instrumental support and perceived social support [62]. However, length of residency in a neighbourhood could improve social support because an individual has more time to form meaningful social connections. Despite this, in Canada, the majority of those living in housing below standards are more likely to be from the lowest income groups, unemployed adults, new immigrants, and those belonging to a visible minority group [66]. Furthermore, with inflated housing prices, it forces these underprivileged persons to relocate often which can impair adequate prenatal care and perpetuate social deprivation [67]. This indicates that other SES indicators such as race, income, and employment are potential mediators between residential stability and adverse health outcomes [68]. This was further supported by our study results as no significant differences were found between infants with mothers residing in the highest and lowest quintile of residential instability and risk of CHD after adjusting for other maternal factors; however, an increasing prevalence of CHD and high residential instability was observed. Moving forward, it is important to evaluate which components of the residential instability index may influence infant CHD outcomes.

The finding regarding the relationship between living in higher ethnic concentration neighbourhoods and lower risk of infant CHD is consistent with the results of prior studies that have found a positive association between immigration and minority status and the risk of CHD among infants [22, 69]. The domain of ethnic concentration measures the concentration of people living in an area who are immigrants and/or who identify as a part of a visible minority group [31]. According to a recent report, over 3.8 million Ontarians (29.3%) were identified as a member of a visible minority group and the majority of visible minorities (2.3 millions of these 3.8 million) were immigrants between 2001–2016 [70]. As such, it is highly likely that this observed relationship is at least partly due to the healthy immigrant effect, which states that recent immigrant populations tend to be healthier compared to the populations born in Canada [71–73]. In addition, an American study found that the overall incidence rate of CHD amongst infants born to mothers belonging to racial minority groups was higher compared to White mothers [23]. The heterogeneity of different racial minority groups may also play a role as previous studies have shown that the risk of CHD varies according to the different minority groups even after adjusting for SES factors and other covariates [17, 19, 23]. For instance, we previously found that the overall CHD incidence rate was lower among Asian infants as compared to Black infants in Ontario [19]. However, we were not able to differentiate the effects of race and immigrants in this study since we used a community level composite measurement. More studies are needed to examine pregnant people’s immigrant status and race with regard to CHD in order to fully understand the mechanism.

Comparably, sex-differences have been previously identified in relation to CHD severity and mortality rates [15, 74, 75]. Some studies have shown that male infants have a 5% higher risk of mortality compared to female infants [74, 76]. This finding has provided more evidence to support other researchers’ hypothesis that, compared to female infants, male infants are born with more severe types of CHD that require early surgical interventions [74, 75]. Despite the sex-disparity in CHD prevalence, we did not find that infant sex significantly modified the association between each of the four dimensions of maternal SES and risk of CHD.

In this study, we found that there were higher termination rates among fetuses diagnosed with CHD. The higher termination rate may be a result of the prenatal diagnosis of CHD. Disadvantaged pregnant individuals might experience barriers to accessing optimal Ontario prenatal health care, including prenatal ultrasound and referral to fetal echocardiography, if the healthcare professionals suspect fetal CHD. Furthermore, some ethnic/religious groups may be less likely to pursue the possibility of pregnancy termination due to cultural and/or religious reasons. Thus, these groups of pregnant individuals in this population may give birth to more infants with CHD compared to their more advantaged peers. Future researchers should study the associations between CHD, prenatal care, and pregnancy termination.

Furthermore, we also observed that mothers with obesity had higher odds of having an infant with CHD compared to mothers without obesity. This finding may suggest that maternal obesity is associated with gestational diabetes and may increase the risk of congenital heart disease in the fetus/offspring. This disparity on the incidence of congenital heart disease between disadvantaged and more advantaged pregnant individuals needs further investigation as well.

There were many strengths to this study. This is the first study that used the ON_Marg as a proxy for maternal SES to further examine the relationship between SES and risk of infant CHD. The ON_Marg incorporated four different perspectives of SES to better capture the complexity of SES and allowed for a more detailed exploration of which SES factors are more closely tied to a higher risk of CHD development. Furthermore, all singleton births from 2012 to 2018 in Ontario, Canada were included to produce a large sample size to improve the precision of the study results and reduce the chance of selection bias and random errors. Lastly, this study controlled for many known confounders between the maternal SES and infant CHD pathway such as maternal age, pre-existing maternal conditions, and rural residency to better analyze the associations between specific SES factors and infant CHD outcomes.

Despite these strengths, there were also a few limitations to this study. First, approximately 10% of the data from the ON_Marg index was missing [31]. Moreover, data for pregnancy termination or spontaneous loss before gestational age of 20 weeks is not available in the BORN database and was therefore not included. In addition, there may be potential misclassification of CHD diagnosis as the data was gathered through passive surveillance. Furthermore, despite controlling for many confounders, residual confounding remains within our analysis since data on the family history of CHD and genetic factors were not considered due to limitations of the data. Lastly, the ON_Marg used a small area DA-based SES indicators; there is a potential for misclassification of SES although DA-based SES has been widely used to estimate individual SES when individual SES information is lacking [20]. Generalisability may also be limited as structural factors contributing to area level measures, such as percent unemployed or literacy, may vary across jurisdictions and reflect policy matters. Moreover, the use of composite area level exposures, while providing evidence of community factors related to CHDs, is limited for identifying specific causal pathways and preventive strategies. Hence, future studies should investigate both individual and community level SES indicators and evaluate the impacts on the CHD burden.

## Conclusions

In summary, this study found that residing in areas of high material deprivation and low ethnic concentration can increase the risk of infant CHD. This suggests that health and public health interventions and policies should be aimed towards families living in poverty to decrease the perpetuating SES inequity gap. Further research is required to investigate the mechanisms behind the protective effect observed with immigration and visible minority status and SES.

### Disclaimer

Parts of this material are based on data and information compiled and provided by CIHI. However, the analyses, conclusions, opinions, and statements expressed herein are those of the author(s), and not necessarily CIHI.

## Supplementary Information

**Additional file 1.**

## Data Availability

The data analyzed for this study are held securely at the prescribed registry, BORN Ontario. Data sharing regulations prevent these data from being made available publicly due to the personal health information in the datasets. Enquiries regarding BORN data must be directed to BORN Ontario (Science@BORNOntario.ca).

## Abbreviations

CHD: Congenital heart disease
ON_Marg: Ontario Marginalization Index
SES: Socioeconomic status
DA: Dissemination area
OR: Odds ratio
aOR: Adjusted odds ratio
ART: Assisted reproductive technology
CIHI: Canadian Institute for Health Information
BORN: Better Outcomes Registry & Network
BIS: BORN information system
BMI: Body mass index

## References

[1] Sun RR, Liu M, Lu L, Zheng Y, Zhang P. Congenital Heart Disease: Causes, Diagnosis, Symptoms, and Treatments. Cell Biochem Biophys. 2015;72(3):857–60. Available from: https://pubmed.ncbi.nlm.nih.gov/25638345/10.1007/s12013-015-0551-6 25638345

[2] Zimmerman MS, Smith AGC, Sable CA, Echko MM, Wilner LB, Olsen HE, et al. Global, regional, and national burden of congenital heart disease, 1990–2017: a systematic analysis for the Global Burden of Disease Study 2017. Lancet Child Adolesc Heal. 2020;4(3):185–200. Available from: http://www.thelancet.com/article/S235246421930402X/fulltext 10.1016/S2352-4642(19)30402-X PMC7645774 31978374

[3] Liu Y, Chen S, Zühlke L, Black GC, Choy MK, Li N (2019). Global birth prevalence of congenital heart defects 1970–2017: Updated systematic review and meta-analysis of 260 studies. Int J Epidemiol.

[4] Van Der Linde D, Konings EEM, Slager MA, Witsenburg M, Helbing WA, Takkenberg JJM (2011). Birth prevalence of congenital heart disease worldwide: A systematic review and meta-analysis. J Am Coll Cardiol.

[5] Kaur A, Hornberger LK, Fruitman D, Ngwezi DP, Chandra S, Eckersley LG. Trends in the Prenatal Detection of Major Congenital Heart Disease in Alberta From 2008–2018. J Obstet Gynaecol Canada. 2022;44(8):895–900. Available from: http://www.jogc.com.proxy1.lib.uwo.ca/article/S1701216322003395/fulltext 10.1016/j.jogc.2022.03.020 35513257

[6] Pruthi V, Thakur V, Jaeggi E, Rowbottom L, Naguleswaran K, Ryan G, et al. Impact of Planned Delivery on the Perinatal Outcome of Term Fetuses with Isolated Heart Defects. J Obstet Gynaecol Canada. 2022;44(8):901–7. Available from: http://www.jogc.com/article/S1701216322003449/fulltext 10.1016/j.jogc.2022.03.022 35598862

[7] Liu S, Joseph KS, Luo W, León JA, Lisonkova S, Van Den Hof M (2016). Effect of folic acid food fortification in Canada on congenital heart disease subtypes. Circulation.

[8] Statistics Canada. Births, 2020. The Daily. 2021 [cited 2022 Jul 26]. Available from: https://www150.statcan.gc.ca/n1/daily-quotidien/210928/dq210928d-eng.htm

[9] Mackie AS, Tran DT, Marelli AJ, Kaul P. Cost of Congenital Heart Disease Hospitalizations in Canada: A Population-Based Study. Can J Cardiol. 2018;33(6):792–8. Available from: 10.1016/j.cjca.2017.01.024 10.1016/j.cjca.2017.01.024 28457736

[10] Islam S, Kaul P, Tran DT, Mackie AS (2018). Health Care Resource Utilization Among Children With Congenital Heart Disease: A Population-Based Study. Can J Cardiol..

[11] Williams K, Carson J, Lo C (2019). Genetics of Congenital Heart Disease. Biomolecules..

[12] Jenkins KJ, Correa A, Feinstein JA, Botto L, Britt AE, Daniels SR (2007). Noninherited risk factors and congenital cardiovascular defects: Current knowledge - A scientific statement from the American Heart Association Council on Cardiovascular Disease in the Young. Circulation.

[13] Wen SSW, Miao Q, Taljaard M, JL-J, 2020 undefined, Lougheed J, et al. Associations of assisted reproductive technology and twin pregnancy with risk of congenital heart defects. 2020;174(5):446–54. Available from: https://jamanetwork.com/journals/jamapediatrics/fullarticle/2761809 10.1001/jamapediatrics.2019.6096 PMC7042937 32091547

[14] Dadvand P, Rankin J, Rushton S, Pless-Mulloli T. Association between maternal exposure to ambient air pollution and congenital heart disease: A register-based spatiotemporal analysis. Am J Epidemiol. 2011;173(2):171–82. Available from: https://pubmed.ncbi.nlm.nih.gov/21123851/10.1093/aje/kwq342 PMC3011953 21123851

[15] Rothman KJ, Fyler DC. Sex, birth order, and maternal age characteristics of infants with congenital heart defects. Am J Epidemiol. 1976;104(5):527–34. Available from: https://pubmed.ncbi.nlm.nih.gov/984027/10.1093/oxfordjournals.aje.a112326 984027

[16] Agay-Shay K, Friger M, Linn S, Peled A, Amitai Y, Peretz C. Air pollution and congenital heart defects. Environ Res. 2013;124:28–34. Available from: https://pubmed.ncbi.nlm.nih.gov/23623715/10.1016/j.envres.2013.03.005 23623715

[17] Davey B, Sinha R, Lee JH, Gauthier M, Flores G. Social determinants of health and outcomes for children and adults with congenital heart disease: a systematic review. Pediatr Res. 2020;89(2):275–94. Available from: https://www.nature.com/articles/s41390-020-01196-6 10.1038/s41390-020-01196-6 33069160

[18] Nashed LM, O’Neil J. The impact of socioeconomic status and race on the outcomes of congenital heart disease. Curr Opin Cardiol. 2022;37(1):86–90. Available from: https://journals-lww-com.proxy.queensu.ca/co-cardiology/Fulltext/2022/01000/The_impact_of_socioeconomic_status_and_race_on_the.14.aspx 10.1097/HCO.0000000000000928 34698665

[19] Miao Q, Dunn S, Wen SW, Lougheed J, Maxwell C, Reszel J, et al. Association of maternal socioeconomic status and race with risk of congenital heart disease: a population-based retrospective cohort study in Ontario, Canada. BMJ Open. 2022;12(2):e051020. Available from: https://bmjopen.bmj.com/content/12/2/e051020 10.1136/bmjopen-2021-051020 PMC8808396 35105571

[20] Miao Q, Dunn S, Wen SW, Lougheed J, Sharif F, Walker M (2022). Associations of congenital heart disease with deprivation index by rural-urban maternal residence: a population-based retrospective cohort study in Ontario, Canada. BMC Pediatr..

[21] Yu D, Feng Y, Yang L, Da M, Fan C, Wang S (2014). Maternal socioeconomic status and the risk of congenital heart defects in offspring: A meta-analysis of 33 studies. PLoS ONE.

[22] Miao Q, Dunn S, Wen SW, Lougheed J, Reszel J, Lavin Venegas C (2021). Neighbourhood maternal socioeconomic status indicators and risk of congenital heart disease. BMC Pregnancy Childbirth..

[23] Knowles RL, Ridout D, Crowe S, Bull C, Wray J, Tregay J (2017). Ethnic and socioeconomic variation in incidence of congenital heart defects. Arch Dis Child..

[24] Leifheit KM, Schwartz GL, Pollack CE, Edin KJ, Black MM, Jennings JM (2020). Severe Housing Insecurity during Pregnancy: Association with Adverse Birth and Infant Outcomes. Int J Environ Res Public Health..

[25] Deguen S, Kihal W, Jeanjean M, Padilla C, Zmirou-Navier D (2016). Neighborhood Deprivation and Risk of Congenital Heart Defects, Neural Tube Defects and Orofacial Clefts: A Systematic Review and Meta-Analysis. PLoS ONE.

[26] Galobardes B, Shaw M, Lawlor DA, Lynch JW, Smith GD (2006). Indicators of socioeconomic position (part 1). J Epidemiol Community Health.

[27] Galobardes B, Shaw M, Lawlor DA, Lynch JW, Smith GD. Indicators of socioeconomic position (part 2). J Epidemiol Community Health. 2006;60(2):95–101. Available from: https://pubmed.ncbi.nlm.nih.gov/16415256/10.1136/jech.2004.028092 PMC2566160 16415256

[28] Conway DI, McMahon AD, Brown D, Leyland AH. Measuring socioeconomic status and inequalities. Reducing Soc inequalities cancer Evid priorities Res. 2019 [cited 2022 Jul 26]; Available from: https://www.ncbi.nlm.nih.gov/books/NBK566205/33534498

[29] Herring J. Summary Measures of Socioeconomic Inequalities in Health. 2013 [cited 2022 Jul 26]; Available from: www.publichealthontario.ca

[30] Australian Bureau of Statistics. 2033.0.55.001 - Census of Population and Housing: Socio-Economic Indexes for Areas (SEIFA), Australia, 2016. 2018 [cited 2022 Sep 30]. Available from: https://www.abs.gov.au/ausstats/abs@.nsf/mf/2033.0.55.001

[31] Matheson F, Moloney G, van Ingen T (2016). User Guide: 2016 Ontario Marginalization Index.

[32] Matheson FI, Dunn JR, Smith KLW, Moineddin R, Glazier RH (2012). Development of the Canadian Marginalization Index: a new tool for the study of inequality. Can J Public Health.

[33] Davis LE, Coburn NG, Hallet J, Earle CC, Liu Y, Myrehaug S (2020). Material deprivation and access to cancer care in a universal health care system. Cancer..

[34] Zygmunt A, Kendall CE, James P, Lima I, Tuna M, Tanuseputro P (2020). Avoidable Mortality Rates Decrease but Inequity Gaps Widen for Marginalized Neighborhoods: A Population-Based Analysis in Ontario, Canada from 1993 to 2014. J Community Health..

[35] Nishat F, Lunsky Y, Tarasoff LA, Brown HK (2022). Prenatal Care Adequacy Among Women With Disabilities: A Population-Based Study. Am J Prev Med.

[36] Anderson LN, Fatima T, Shah B, Smith BT, Fuller AE, Borkhoff CM (2022). Income and neighbourhood deprivation in relation to obesity in urban dwelling children 0–12 years of age: a cross-sectional study from 2013 to 2019. J Epidemiol Community Heal..

[37] Sprague AE, Sidney D, Darling EK, Wagner V, Soderstrom B, Rogers J (2018). Outcomes for the First Year of Ontario’s Birth Center Demonstration Project. J Midwifery Womens Health..

[38] Dunn S, Sprague A, Fell D, Dy J, Harrold J, Lamontagne B (2013). The Use of a Quality Indicator to Reduce Elective Repeat Caesarean Section for Low-Risk Women Before 39 Weeks’ Gestation: The Eastern Ontario Experience. J Obstet Gynaecol Can.

[39] Miao Q, Fell DB, Dunn S, Sprague AE (2019). Agreement assessment of key maternal and newborn data elements between birth registry and Clinical Administrative Hospital Databases in Ontario. Canada Arch Gynecol Obstet.

[40] Dunn S, Lanes A, Sprague AE, Fell DB, Weiss D, Reszel J (2019). Data accuracy in the Ontario birth Registry: A chart re-abstraction study. BMC Health Serv Res..

[41] Dunn S, Sprague AE, Grimshaw JM, Graham ID, Taljaard M, Fell D (2016). A mixed methods evaluation of the maternal-newborn dashboard in Ontario: Dashboard attributes, contextual factors, and facilitators and barriers to use: A study protocol. Implement Sci..

[42] CIHI. Discharge Abstract Database Metadata (DAD). [cited 2018 Oct 22]. Available from: https://www.cihi.ca/en/discharge-abstract-database-metadata

[43] CIHI. National Ambulatory Care Reporting System metadata (NACRS) | CIHI. [cited 2022 Aug 15]; Available from: https://www.cihi.ca/en/national-ambulatory-care-reporting-system-metadata-nacrs

[44] The Postal CodeOM Conversion File. Statistics Canada. [cited 2018 May 10]. Available from: http://www.statcan.gc.ca/pub/92-154-g/2016001/section03-eng.htm

[45] Statistics Canada. 2016 Canadian Census. [cited 2020 Dec 15]. Available from: https://www12.statcan.gc.ca/census-recensement/2016/dp-pd/index-eng.cfm

[46] Statistics Canada. Dictionary, Census of Population, 2016 - Dissemination area (DA). 2016. Available from: https://www12.statcan.gc.ca/census-recensement/2016/ref/dict/geo021-eng.cfm

[47] Wu W, He J, Shao X (2020). Incidence and mortality trend of congenital heart disease at the global, regional, and national level, 1990–2017. Med (United States)..

[48] Giang KW, Mandalenakis Z, Fedchenko M, Eriksson P, Rosengren A, Norman M, Hanséus K, Dellborg M (2023). Congenital heart disease: changes in recorded birth prevalence and cardiac interventions over the past half-century in Sweden. Eur J Prev Cardiol.

[49] Hoffman JIE (1995). Incidence of Congenital Heart Disease: II. Prenatal Incidence Pediatr Cardiol.

[50] Marelli AJ, Mackie AS, Ionescu-ittu R, Rahme E (2007). Congenital Heart Disease in the General Population. Circulation..

[51] Agha MM, Glazier RH, Moineddin R, Moore AM, Guttmann A (2011). Socioeconomic status and prevalence of congenital heart defects : does universal access to health care system eliminate the gap ?. Birth Defects Res A Clin Mol Teratol..

[52] Li X, Sundquist J, Hamano T, Zöller B, Sundquist K. Neighbourhood Deprivation, Individual-Level and Familial-Level Socio-demographic Factors and Risk of Congenital Heart Disease: A Nationwide Study from Sweden. Int J Behav Med. 2016;23(1):112–20. Available from: https://pubmed.ncbi.nlm.nih.gov/25929332/10.1007/s12529-015-9488-9 PMC4808140 25929332

[53] Peyvandi S, Baer RJ, Chambers CD, Norton ME, Rajagopal S, Ryckman KK (2020). Environmental and socioeconomic factors influence the live-born incidence of congenital heart disease: a population-based study in California. J Am Heart Assoc..

[54] Stirbu I, Kunst AE, Mielck A, MacKenbach JP. Inequalities in utilisation of general practitioner and specialist services in 9 European countries. BMC Health Serv Res. 2011;11(1):1–8. Available from: 10.1186/1472-6963-11-288 10.1186/1472-6963-11-288 PMC3221557 22040155

[55] Arpey NC, Gaglioti AH, Rosenbaum ME (2017). How socioeconomic status affects patient perceptions of health care: a qualitative study. J Prim Care Community Health..

[56] Ingrid Goh Y, Bollano E, Einarson TR, Koren G. Prenatal multivitamin supplementation and rates of congenital anomalies: a meta-analysis. J Obstet Gynaecol Can. 2006;28(8):680–9. Available from: https://pubmed.ncbi.nlm.nih.gov/17022907/10.1016/S1701-2163(16)32227-7 17022907

[57] Wong P, Denburg A, Dave M, Levin L, Morinis JO, Suleman S, et al. Early life environment and social determinants of cardiac health in children with congenital heart disease. Paediatr Child Health. 2018;23(2):92–5. Available from: https://academic.oup.com/pch/article/23/2/92/4587558 10.1093/pch/pxx146 PMC5905484 29686491

[58] Kessler D, Hevenstone D. The impact of unemployment benefits on birth outcomes: Quasi-experimental evidence from European linked register data. PLoS One. 2022;17(3):e0264544. Available from: 10.1371/journal.pone.0264544https://journals.plos.org/plosone/article?id=10.1371/journal.pone.0264544 10.1371/journal.pone.0264544 PMC8890730 35235603

[59] Brand JE (2015). The Far-Reaching Impact of Job Loss and Unemployment. Annu Rev Sociol..

[60] Provencher C, Milan A, Hallman S, Aoust CD’. Fertility: Overview, 2012 to 2016. 2015 [cited 2022 Aug 9]; Available from: www.statcan.gc.ca

[61] Bonnes M, Carrus G (2004). Environmental Psychology. Overview Encycl Appl Psychol Three-Volume Set.

[62] Turney K, Harknett K (2010). Neighborhood Disadvantage, Residential Stability, and Perceptions of Instrumental Support among New Mothers. J Fam Issues..

[63] Stingone JA, Luben TJ, Daniels JL, Fuentes M, Richardson DB, Aylsworth AS, et al. Maternal exposure to criteria air pollutants and congenital heart defects in offspring: Results from the National Birth Defects Prevention Study. Environ Health Perspect. 2014;122(8):863–72. Available from: 10.1289/ehp.1307289 PMC4123026 24727555

[64] Harville EW, Rabito FA. Housing conditions and birth outcomes: The National Child Development Study. Environ Res. 2018;161:153–7. Available from: https://pubmed.ncbi.nlm.nih.gov/29149678/10.1016/j.envres.2017.11.012 29149678

[65] Swope CB, Hernández D (2019). Housing as a determinant of health equity: A conceptual model. Soc Sci Med.

[66] Public Health Agency of Canada. Key Health Inequalities in Canada (A National Portrait). Pan-Canadian Public Heal Netw. 2018. https://www.canada.ca/en/public-health/services/publications/science-research-data/key-health-inequalities-canada-national-portrait-executive-summary.html.

[67] Persad RA. Spatio-temporal analysis of mental illness and the impact of marginalization-based factors: a case study of Ontario, Canada. 2020;26(3):237–50. Available from: 10.1080/19475683.2020.1791251

[68] Nowak AL, Giurgescu C. The Built Environment and Birth Outcomes: A Systematic Review. MCN Am J Matern Nurs. 2017;42(1):14–20. Available from: https://pubmed.ncbi.nlm.nih.gov/27755063/10.1097/NMC.0000000000000299 27755063

[69] Hoyt AT, Shumate CJ, Canfield MA, Le M, Ramadhani T, Scheuerle AE. Selected acculturation factors and birth defects in the National Birth Defects Prevention Study, 1997–2011. Birth Defects Res. 2019;111(10):598–612. Available from: 10.1002/bdr2.1494 10.1002/bdr2.1494 31021057

[70] Statistics Canada. Census Profile, 2016 Census - Ontario [Province] and Canada [Country] [Internet]. [cited 2022 Aug 9]. Available from: https://www12.statcan.gc.ca/census-recensement/2016/dp-pd/prof/details/page.cfm?Lang=E&Geo1=PR&Code1=35&Geo2=PR&Code2=01&Data=Count&SearchText=35&SearchType=Begins&SearchPR=01&B1=All&Custom=&TABID=3

[71] De Maio FG, Kemp E. The deterioration of health status among immigrants to Canada. 2009;5(5):462–78. Available from: 10.1080/17441690902942480 10.1080/17441690902942480 19513909

[72] Vang ZM, Sigouin J, Flenon A, Gagnon A (2017). Are immigrants healthier than native-born Canadians? A systematic review of the healthy immigrant effect in Canada. Ethn Heal.

[73] Lu C, Ng E. Health Reports Healthy immigrant effect by immigrant category in Canada. 2019 [cited 2022 Aug 9]; Available from: 10.25318/82-003-x201900400001-eng 10.25318/82-003-x201900400001-eng 30994921

[74] Marelli A, Gauvreau K, Landzberg M, Jenkins K. Sex differences in mortality in children undergoing congenital heart disease surgery: a United States population-based study. Circulation. 2010;122(11 Suppl). Available from: https://pubmed.ncbi.nlm.nih.gov/20837919/10.1161/CIRCULATIONAHA.109.928325 20837919

[75] Šamánek M. Boy:girl ratio in children born with different forms of cardiac malformation: A population-based study. Pediatr Cardiol. 1994;15(2):53–7. Available from: 10.1007/BF00817606 10.1007/BF00817606 7997413

[76] Report of the New England Regional Infant Cardiac Program. Pediatrics. 1980;65(2 Pt 2):375–461. 7355042

