# Supplementary material for: Association between maternal marginalization and infants born with congenital heart disease in Ontario Canada
Source: BMC Public Health. 2023 Apr 28;23:790. doi: 10.1186/s12889-023-15660-5 (PMC10142402; doi:10.1186/s12889-023-15660-5)
Supplement: Supplementary file 1 — Additional file 1. [file 12889_2023_15660_MOESM1_ESM.docx]

**Appendix A.** Ontario Marginalization Index Dimension Descriptions

Table S1. Ontario Marginalization Index Dimensions and their Corresponding Indicators and Measurements

| **Dimension** | **Indicators** | **Definition** | **Notes** |
| --- | --- | --- | --- |
| Material Deprivation | Proportion of individuals aged 20+ without a high school diploma | (# person aged 20+ without a certificate, diploma, or degree)/ (total # persons aged 20+) | Education Attainment |
|  | Proportion of families who are single parents | (# single parent families)/(total # census families) | Family Structure |
|  | Proportion of total income that are government transfer payments for individuals aged 15+ | (median $ from government transfer for census families)/(median $ from all income sources for census family) | Income |
|  | Proportion of individuals considered as low income (earning less than low-income cut-off) | (# people living in families earing less than the after-tax low income measure)/(total # people) | Income |
|  | Proportion of households residing in dwellings that need major repairs | (# residential households in poor/fair conditions)/(total # of residential households) | Quality of Housing |
| Dependency | Proportion of the population who are 65 years or older | (# people aged 65+)/(total population) | Work not compensated |
|  | Dependency ratio | (total population aged 0-14 and 65+)/(total population aged 15-64) | Adults taking care of households |
|  | Proportion of population aged 15+ who are not participating in the workforce | (# people aged 0-14 and 65+)/(# population aged 15-64) | Work not compensated |
| Ethnic Concentration | Proportion of population who are recent immigrants (≤5 years) | (# people who came to Canada in the past 5 years)/(total Canadian population) | Immigrants |
|  | Proportion of population who self-identify as visual minority | (# people belonging to visible minority and migrated to Canada between 1985 and 2016 –based on country of birth, first language, and surname)/(total population) | Minorities |
| Residential instability | Proportion of population living alone | (# living alone)/(total population) | Family Characteristic |
|  | Proportion of dwellings that are not owned | (# residential households occupied by owner of property)/(total # residential households) | Type of residential accommodation |
|  | Average number of person per dwelling | Calculated by Statistics Canada | Density of residential accommodation |
|  | Proportion of dwellings that are apartment buildings | (# of residential households with multi-unit property codes)/(total # of residential households) | Type of residential accommodation |
|  | Proportion of population who moved within the past 5 years | # of people with recorded postal code on 2016 index date of July 1st that does not match their postal code on 2011 index date)/ (# people with a valid postal code at index date) | Housing Stability |
|  | Proportion of population who are not youth (5-15 year old) | (# aged 5-15)/(total population) | Family Characteristic |
|  | Proportion of population who are single/divorced/widowed | (# married or common-law)/(total population) | Family Characteristic / Family Stability |

Source: Matheson F, Moloney G, van Ingen T. User Guide: 2016 Ontario Marginalization Index. Ontario Agency Heal Prot Promot (Public Heal Ontario).

**Appendix B.** CHD definitions and classifications

**Congenital heart disease coding using ICD-10-CA**

**a) Overall congenital heart disease (CHD)**

| Including Q20 toQ23 | |  |  |  |  |  |  |  |
| --- | --- | --- | --- | --- | --- | --- | --- | --- |
| Q20 | Congenital malformations of cardiac chambers and connections | | | | | |  |  |
| Q21 | Congenital malformations of cardiac septa | | | |  |  |  |  |
| Q22 | Congenital malformations of pulmonary and tricuspid valves | | | | |  |  |  |
| Q23 | Congenital malformations of aortic and mitral valves | | | | |  |  |  |
| Q24 | Other congenital malformations of heart | | |  |  |  |  |  |
| Including Q24.0, Q24.8, Q24.9, Q24.2 to Q24.5 | | | | |  |  |  |  |
| Q25 Congenital malformations of great arteries, including Q25.1 to Q25.9 | | | | | | | | |
| Q26 Congenital malformations of great veins, including Q26.0 to Q26.4 | | | | | | | |  |

**b) Severe CHD**

Q20.0, Q20.1, Q20.3, Q20.4, Q20.6, Q21.2-Q21.4, Q22.0. Q22.4-Q22.6, Q23.0, Q23.2, Q23.4, Q23.5, Q24.8, Q25.1, Q25.2, Q25.4, Q26.2, Q26.3

**c) Single ventricle congenital heart disease**

Q20.4, Q22.4, Q22.6, Q23.4, Q25.1
